# The Management of Interstitial Lung Disease in the ICU: A Comprehensive Review

**DOI:** 10.3390/jcm13226657

**Published:** 2024-11-06

**Authors:** Zehra Dhanani, Rohit Gupta

**Affiliations:** 1 Thoracic Medicine and Surgery, Temple University Hospital, Philadelphia, PA 19140, USA; zehra.dhanani@tuhs.temple.edu; 2 Thoracic Medicine and Surgery, Lewis Katz School of Medicine at Temple University, Philadelphia, PA 19140, USA

**Keywords:** interstitial lung disease, pulmonary fibrosis, acute exacerbation, mechanical ventilation, lung transplantation

## Abstract

Interstitial lung disease (ILD) encompasses a diverse group of parenchymal lung diseases characterized by varying degrees of inflammation and/or fibrosis. Patients with ILD frequently require hospitalization, with many needing intensive care unit (ICU) admission, most often due to respiratory failure. The diagnosis and management of ILD in the ICU present unique challenges. Diagnosis primarily relies on chest CT imaging to identify fibrosis and inflammation. Acute exacerbations, whether in idiopathic pulmonary fibrosis (IPF) or non-IPF ILD, require careful evaluation of potential triggers and differential diagnoses. Bronchoalveolar lavage may provide valuable information, such as the identification of infections, but carries risks of complications. Biopsies, whether transbronchial or surgical, can also be informative but pose significant procedural risks. Corticosteroids are the cornerstone of treatment for acute exacerbations of IPF, with higher doses potentially benefiting non-IPF ILD. Additional immunosuppressive agents may be used in cases with evidence of inflammation. Oxygen supplementation, particularly with high-flow nasal cannula, is often employed to manage severe hypoxemia, while noninvasive ventilation can be useful for worsening hypoxemia and/or hypercapnia. When mechanical ventilation is used, it is recommended to target low tidal volumes to minimize lung injury; high PEEP may be less effective and even associated with increased mortality. Prone positioning can improve oxygenation in severely hypoxemic patients. In addition to ventilatory strategies, careful fluid management and addressing concomitant pulmonary hypertension are essential components of care. Extracorporeal membrane oxygenation is a high-risk intervention reserved for the most severe cases. Lung transplantation may be considered for end-stage ILD patients in the ICU, with outcomes dependent on the urgency of transplantation and the patient’s overall condition. Managing ILD in the ICU requires a multidisciplinary approach, and despite recent advances, mortality remains high, emphasizing the need for continued research and individualized treatment strategies.

## 1. Introduction

Interstitial lung disease (ILD) comprises a heterogeneous group of parenchymal lung diseases characterized by varying degrees of inflammation and/or fibrosis. Based on radiographic features, ILD can be broadly classified into two main groups: the fibrosis-predominant group and the inflammation- or cellular-predominant group [1,2]. Idiopathic pulmonary fibrosis (IPF), a classic example of fibrosis-predominant ILD, is characterized by the usual interstitial pneumonia pattern on imaging [3]. IPF is associated with a poor prognosis, with an estimated 5-year survival rate of approximately 50% [4]. In a large IPF registry study by Kim et al. involving over 1000 patients, 56.7% experienced at least one hospitalization during a two-year follow-up period, and 21.1% required ventilatory support [5].

Among ILD patients, admission to the intensive care unit (ICU) is most often necessitated by acute hypoxemic respiratory failure, frequently in the setting of an acute exacerbation. However, other etiologies, such as extra-parenchymal causes including pneumothorax, pleural effusion, and pulmonary embolism, must be ruled out [6,7] (Table 1). Exacerbations are particularly well documented in patients with IPF, where the incidence rate of exacerbations ranges from 4% to 20%, with those requiring ICU care generally exhibiting shorter survival compared to non-exacerbating patients [4,7]. By contrast, patients with non-IPF ILD typically experience fewer exacerbations and have lower mortality rates [8]. Factors such as poor baseline lung function, as indicated by reduced forced vital capacity (FVC) and diffusion capacity of the lung for carbon monoxide (DLCO), older age, and radiographic patterns of honeycombing are associated with an increased risk of exacerbations and poor prognosis [9,10,11].

Given the scarcity of comprehensive research addressing the care of ILD patients in the ICU and the high morbidity and mortality associated with ICU admission in this population, consolidating current evidence is crucial for guiding clinicians in optimizing management strategies. Furthermore, a thorough understanding of these management strategies is essential, particularly as more end-stage ILD patients undergo rapid evaluation for lung transplantation while in the ICU. This knowledge can increase the likelihood of successful transplantation and improve survival outcomes. Despite the importance of these clinical issues, much of the available data stem from observational studies or are extrapolated from general ICU management principles. This review aims to address these gaps by providing a comprehensive overview of ILD management in the ICU, focusing on diagnostic and therapeutic considerations critical for improving outcomes, and highlighting potential avenues for future research.

### 1.1. The Diagnosis and Evaluation of Patients with ILD in the ICU

**Figure 1:**
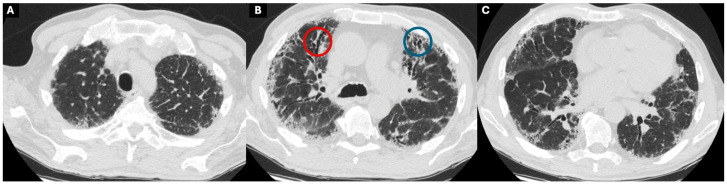
Chest CT images of a patient with fibrosis-predominant ILD showing honeycombing (image (**B**)), traction bronchiectasis (image (**B**)), and interstitial thickening. Image (**A**–**C**) show cross-sectional views of chest CT images of a patient with fibrosis-predominant ILD. Red circle shows traction bronchiectasis and blue circle shows honeycombing.

**Figure 2:**
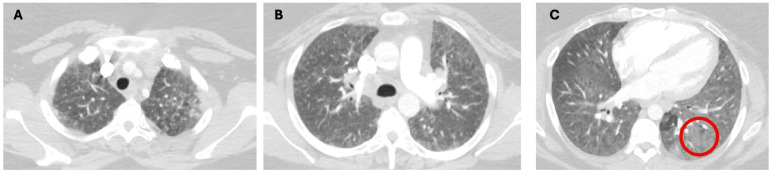
Chest CT images of a patient with inflammation-predominant ILD showing ground-glass opacities (image (**C**)). Image (**A**–**C**) show cross-sectional views of chest CT images of a patient with inflammation-predominant ILD. Red circle shows an area of ground-glass opacities.

Diagnosing ILD in the ICU presents unique challenges, as this patient population may include both those who are newly diagnosed with ILD during their ICU stay and those with a pre-existing diagnosis of ILD. The diagnosis of ILD primarily relies on imaging findings, particularly high-resolution CT (HRCT) scans. HRCT features indicative of fibrosis include reticulations, interstitial thickening, traction bronchiectasis, and honeycombing [3] (Figure 1). In contrast, inflammation may be characterized by ground-glass opacities (GGOs) [13] (Figure 2). However, it is important to note that GGOs are nonspecific and can represent the intra-alveolar filling process of any etiology such as infection, fluid accumulation, and cellular infiltration [14]. Some imaging features might point toward a diagnosis of a specific ILD, but distinguishing between different ILDs involves combining these imaging characteristics and distribution, along with a thorough history and laboratory investigations, including but not limited to an autoimmune panel, drug use, and exposure history [1]. A multidisciplinary approach can be particularly valuable in managing complex cases and refining the diagnostic process [15].

**Figure 3:**
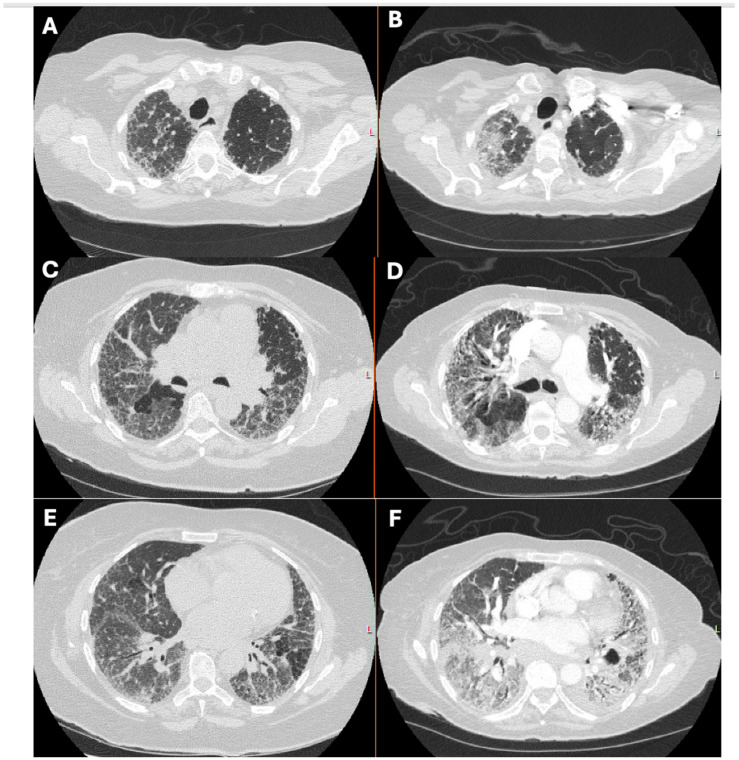
Chest CT images of a patient with ILD at baseline and with an acute exacerbation (a few months after). The images (**A**,**C**,**E**) show the baseline disease involving interstitial thickening, reticulations, and mosaicism, while the images (**B**,**D**,**F**) show pronounced ground-glass opacities and increased interstitial infiltrates, indicative of an exacerbation when the patient was in the ICU.

In the context of a pre-existing ILD diagnosis, an acute exacerbation of ILD (AE-ILD) should be considered a possible cause of acute decompensation [6]. Comparing baseline HRCT findings taken during the stable disease state with those obtained during acute decompensation can provide crucial insights into disease progression, superimposed inflammation, or other potential causes of decline (Figure 3). An acute exacerbation of IPF (AE-IPF) is defined as acute respiratory decompensation occurring within 30 days, with parenchymal findings of ground-glass opacities (GGOs) or consolidation on CT scans that cannot be fully explained by pulmonary edema [7,16]. Acute exacerbation of non-IPF ILD (AE-non-IPF) is less well defined but follows a similar approach, where extra-parenchymal causes of acute respiratory decompensation, such as pneumothorax and pulmonary embolism, must be ruled out [6]. The overall annual incidence of AE-non-IPF, which is 1–3% for connective tissue disease-related ILD with a nonspecific interstitial pneumonia pattern, tends to be lower compared to that of AE-IPF, which ranges from 4 to 20% [7,8].

These acute exacerbations can be triggered by various factors, such as infections, procedures, and exposure to drugs or toxins [6,7]. Patients with an identified trigger generally have better outcomes compared to those with idiopathic exacerbations, likely because the underlying cause(s) can potentially be treated [17]. Since infections are the most common trigger of AE-ILD, obtaining a sputum sample is crucial. When sputum collection is not feasible, bronchoalveolar lavage (BAL) can be considered an alternative. BAL not only allows for the evaluation of infections but also provides important insights into cellular patterns and cytology, which help narrow down the diagnosis. While BAL is commonly obtained in the outpatient setting, its role in the ICU for diagnosing infections and/or ILD is less well understood [18]. Chang et al. demonstrated that a significant number of ICU patients received a new or alternative ILD diagnosis based on BAL results [19]. Conversely, Arcadu et al. found that only a small proportion of BAL procedures yielded positive results and that the procedure was associated with a high rate of patients requiring mechanical ventilation and reintubation [20]. BAL holds significant value in diagnosing opportunistic infections, such as Pneumocystis jirovecii pneumonia (PJP), where it serves as the gold standard for diagnosis [21]. BAL can also assist in diagnosing noninfectious conditions, such as diffuse alveolar hemorrhage, which is identified by a progressively bloody return on serial aliquot administration. However, BAL is not without risks. In a study of 167 critically ill, mechanically ventilated patients who underwent bronchoscopy with BAL, post-procedural hypoxia and bronchospasm occurred in 29% and 9% of cases, respectively. Notably, only 38% had a respiratory diagnosis, and complications may be higher among ILD patients due to limited respiratory reserve [22]. The decision to perform BAL may hinge on intubation status, as it is more feasible in already intubated patients. Therefore, BAL should be used selectively, with clear diagnostic aims.

Obtaining transbronchial or surgical biopsies may be tempting but carries significant risks for patients with ILD in the ICU. Studies suggest that transbronchial biopsy has variable yield and a high complication rate. A review of 42 patients with unexplained respiratory failure (18% with ILD) who underwent transbronchial lung biopsies reported a 12.5% incidence of pneumothorax and 7.1% of significant bleeding [23,24]. Similarly, while surgical lung biopsy is associated with high mortality, it can provide significant diagnostic yield and lead to changes in management [25,26]. Additionally, as many ILDs progress to fibrosis, biopsy may not always yield meaningful information. Therefore, while biopsy has a role, it should be performed with great caution.

### 1.2. The Treatment of Patients with ILD in the ICU

**Figure 4:**
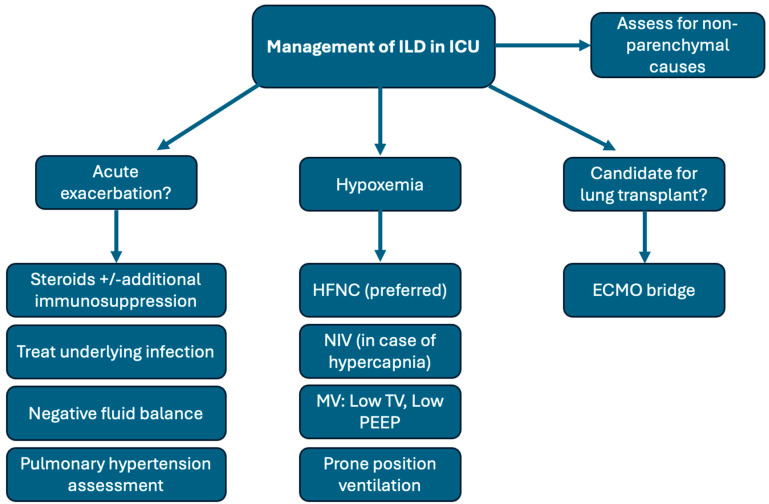
The management of patients with ILD in the ICU. ILD (interstitial lung disease), ICU (intensive care unit), HFNC (high-flow nasal cannula), NIV (noninvasive ventilation), MV (mechanical ventilation), TV (tidal volume), and PEEP (positive end-expiratory pressure). The flowchart outlines a comprehensive approach to managing interstitial lung disease (ILD) in the ICU. Non-parenchymal causes must be ruled out. Acute exacerbations may be managed by using corticosteroids or other immunosuppressives, and careful fluid management, along with pulmonary hypertension assessment/management. For hypoxemia, various oxygenation strategies like HFNC and mechanical ventilation are utilized, tailored to patient needs. In severe cases, candidacy for lung transplantation is considered, potentially utilizing ECMO as a bridge to transplant.

The management of ILD in the ICU is complex, requiring careful consideration of coexisting morbidities. The backbone of treatment includes identifying and addressing the trigger for acute decompensation, assessing the need for immunosuppression, and ensuring appropriate oxygen supplementation. Special attention should be given to the patient’s volume status and right heart function. When applicable, timely initiation of lung transplant evaluation and the potential use of extracorporeal membrane oxygenation (ECMO) should be considered. A multidisciplinary approach is essential to provide comprehensive care for these critically ill patients. A simplified overview of these management strategies is shown in Figure 4.

## 2. Immunosuppression

Corticosteroids remain the mainstay of therapy for AE-IPF. This approach is primarily based on expert opinion, as there are no large randomized controlled trials supporting its use in IPF exacerbations. Guidelines emphasize the anecdotal utility of corticosteroids and the poor prognosis associated with AE-IPF. The usual prescribed dose of corticosteroids is 0.5–1.0 mg/kg per day [3]. However, extra caution is necessary in patients with concomitant infections. Naccache et al. investigated the addition of cyclophosphamide to corticosteroids for treating AE-IPF and found that it increased 3-month all-cause mortality, although not to a statistically significant degree [27]. Observational cohort studies have explored other immunosuppressive agents, such as azathioprine, tacrolimus, rituximab with plasma exchange, and intravenous immunoglobulin (IVIG), which show mixed outcomes [28,29,30]. However, their true efficacy is yet to be determined, and they are not widely used in clinical practice.

There is somewhat stronger evidence for using corticosteroids in the management of acute exacerbations of non-IPF ILD. Jang et al. demonstrated that higher doses of corticosteroids (1–2 mg/kg in divided doses) improved survival in patients with non-IPF ILD compared to those with IPF [31]. Pulse-dose corticosteroids may be employed in patients with severe, rapidly progressive ILD; however, caution should be exercised.

For patients with systemic sclerosis, the Scleroderma Lung Study 1 (SLS 1) showed that cyclophosphamide was effective in improving lung function, dyspnea, and health-related quality of life among patients with systemic sclerosis who had active alveolitis [32]. Similarly, the Scleroderma Lung Study 2 (SLS 2) found that both cyclophosphamide and mycophenolate significantly improved lung function, dyspnea, and other disease parameters, with mycophenolate being better tolerated than cyclophosphamide [33]. Notably, these studies did not include acutely ill patients admitted to the ICU; however, their findings may still be relevant for patients with acute exacerbations, as the patients included in both studies exhibited signs of active ongoing lung inflammation, as evidenced by imaging. Cyclophosphamide was significantly associated with side effects, such as leukopenia, and had a shorter time to withdrawal, which may be particularly concerning for ICU patients predisposed to these adverse effects. However, there are limited data examining these immunosuppressive drugs in the intensive care setting. A small cohort study of patients with severe respiratory failure due to systemic sclerosis and ANCA-associated ILD demonstrated positive outcomes with cyclophosphamide in the ICU [34].

Among other immunosuppressive therapies, azathioprine, once commonly used, has fallen out of favor in systemic sclerosis after its inferiority to cyclophosphamide (and, by extension, mycophenolate) was shown in terms of patient outcomes, including pulmonary function [35]. Rituximab has emerged as a valuable treatment option in systemic sclerosis. The DESIRES study showed that rituximab improved lung function (FVC) in patients with systemic sclerosis, and the EVER-ILD trial found that rituximab combined with mycophenolate delayed lung function decline in patients with ILD with an inflammatory pattern [36,37]. Extrapolating these findings to critically ill patients, Keir et al. reported favorable outcomes with rituximab in a series of eight patients with ILDs associated with various connective tissue diseases [38].

Biologics, such as tocilizumab, have gained popularity over the last decade. Trials in 2016 and 2020 assessed the use of tocilizumab in systemic sclerosis and showed delayed lung function decline (secondary outcome) compared to placebo [39,40]. However, its role in the ICU during acute exacerbations remains unclear, and data in this setting are limited.

In cases of rapidly progressive non-IPF ILD, such as MDA-5 dermatomyositis-associated ILD, there are recommendations for initiating treatment with multiple immunosuppressive agents rather than using a step-up approach [41]. A case series by Matsushita et al. demonstrated remission in all patients treated with a combination of corticosteroids, tacrolimus, and cyclophosphamide [42]. Similarly, rituximab has shown favorable outcomes in patients with rapidly progressive ILD, particularly in those with anti-melanoma differentiation-associated protein 5 (MDA-5) dermatomyositis-associated ILD [43]. The use of IVIG and plasmapheresis may be considered in resistant disease states, depending on the underlying condition; for example, both IVIG and plasmaphereses have been associated with improved survival among patients with anti-MDA-5 dermatomyositis-related ILD [44,45], while plasmapheresis has also been linked to increased mortality among patients with SLE-associated DAH [46]. We agree with the approach suggested by Hallowell et al., where the authors recommend starting with a combination of corticosteroids and another immunosuppressive agent, such as mycophenolate, azathioprine, or tacrolimus, along with IVIG upfront, with the consideration of plasmapheresis in cases where the disease remains refractory, for rapidly progressive ILD with severe symptoms [47].

Another important setting where immunosuppression plays a crucial role is in managing DAH, a significant and often severe complication of inflammatory ILD. High-dose steroids are commonly employed to target the inflammatory component, although their effectiveness in critically ill patients remains uncertain [48,49]. Other immunosuppressive agents, such as rituximab, offer an alternative for refractory cases, with studies showing effectiveness in reducing autoantibody production and controlling disease activity [50]. Plasmapheresis may also be used as adjunctive therapy, particularly in autoimmune conditions, though evidence on its long-term benefit remains limited [51].

## 3. Oxygenation and Ventilation Strategies

Patients with ILD are often hospitalized with respiratory complaints, with hypoxemia being the most common presenting sign [5]. These patients often require increasing amounts of supplemental oxygen to maintain oxygenation, including high-flow nasal cannula (HFNC), noninvasive ventilation (NIV), or mechanical ventilation (MV), necessitating ICU stay.

### 3.1. High-Flow Nasal Cannula (HFNC)

High-flow nasal oxygen provides high flows of noninvasive oxygenation, with FiO_2_ potentially reaching 100%. It also offers the added advantages of a small amount of PEEP and a more comfortable interface [2]. Koyauchi et al. demonstrated that HFNC is effective in improving oxygenation in patients with ILD [52].

### 3.2. Noninvasive Ventilation (NIV)

Noninvasive ventilation is associated with reduced mortality and shorter hospital stays among patients with COPD [53]. However, limited data are available on its utility in ILD patients. Positive end-expiratory pressure (PEEP) in NIV is expected to improve oxygenation, while bilevel pressure assists with ventilation, which could be beneficial for ILD patients experiencing hypoxia and hypercapnia [54]. A meta-analysis by Sanguanwong et al. showed that NIV significantly improved oxygenation, as evidenced by higher P/F ratios compared to conventional oxygenation therapy [55]. It is important to note that there was no difference in survival between patients receiving NIV and those receiving invasive ventilation [56].

A few studies directly comparing NIV to HFNC have shown that NIV improves the P/F ratio more effectively [52,57,58]. However, there was no difference in mortality or the need for invasive ventilation between the two methods [52,57,58,59].

### 3.3. Mechanical Ventilation (MV)

Patients with ILD are at high risk of respiratory decompensation, which often necessitates MV [60]. While mechanical ventilation is frequently used as a rescue strategy in severe respiratory failure, it is associated with high mortality rates among ILD patients. Mollica et al. demonstrated that all patients with IPF who required MV died within the same hospitalization [56]. The high mortality was shown again by Martin et al., who reported a 75% mortality rate among fibrosis-predominant ILD patients requiring MV [60]. MV-related predictors of in-hospital mortality include the inability to achieve early targeted plateau pressure (≥30 cm H_2_O), severe hypoxemia as suggested by a reduced P/F ratio and high initial FiO_2_, and high initial mean airway pressures [60]. However, a recent study by Matsunashi et al. demonstrated a 54% survival rate among patients with fibrosis-predominant ILD who required invasive mechanical ventilation. Although this study had a small sample size, the results suggest that improved ventilatory techniques may help mitigate complications such as barotrauma and volutrauma, potentially enhancing survival outcomes [61].

To avoid ventilator-induced lung injury, various aspects of respiratory physiology must be carefully considered. In patients with pulmonary fibrosis, an imbalance between collagen and elastin deposition in the extracellular matrix leads to increased collagen accumulation, making the lungs stiffer and less compliant in areas affected by fibroblastic foci [62]. Although low tidal volume ventilation (6 mL/kg) has been a cornerstone in managing acute respiratory distress syndrome (ARDS) due to its association with decreased mortality, less is known about the optimal ventilatory strategy for patients with ILD [63]. Nonetheless, experts recommend using low tidal volumes to prevent barotrauma and minimize the proinflammatory effects of lung overstretching, which can lead to the accumulation of inflammatory cells and the release of cytokines and chemokines that exacerbate lung injury [62,64,65].

In contrast to the approach for ARDS, where high positive end-expiratory pressure (PEEP) is used to improve oxygenation and keep alveoli open, high PEEP may not be suitable for ILD patients. Unlike ARDS, ILD patients may not have recruitable alveolar units [64]. In fact, some retrospective studies have linked high PEEP with increased mortality in patients with ILD [66]. There is a theoretical risk of pneumothorax in ILD patients due to high PEEP, resulting from reduced elastance in the non-recruitable lung units and overdistension of the normal lung units, though data supporting this risk are limited.

### 3.4. Prone Position Ventilation

Prone position ventilation is a well-established tool in the management of ARDS, with robust evidence demonstrating improvement in oxygenation and 30-day mortality [67]. However, its utility in ILD patients remains less clear. Lung mechanics in ILD differ from those in ARDS, with reduced compliance due to fibrosis, which limits the recruiting ability of the alveolar units in the prone position. Despite this, limited studies have explored prone positioning in ILD populations. Xu et al. demonstrated that prone position ventilation improved oxygenation and hemodynamics in ILD patients, suggesting potential benefits even in this distinct group [68].

### 3.5. Suggested Approach to Respiratory Support

In our opinion, when managing a patient with ILD and acute hypoxic respiratory failure, the best initial step is to employ HFNC. This method offers high FiO_2_ and flow rates (resulting in a small amount of PEEP), while being generally well tolerated and presenting a lower risk of complications like pneumothorax. For patients who remain hypoxemic or exhibit significant hypercapnia despite HFNC, we believe a trial of NIV is reasonable.

The decision to proceed with intubation is complex and should be individualized based on the specific ILD subtype. Given the particularly poor outcomes associated with mechanical ventilation in IPF, we agree with guidelines recommending against intubation in these patients when clinical judgment indicates minimal chances of recovery and the patient is not a candidate for transplantation. This further highlights the importance of early discussions around goals of care, ideally while the patient can still participate in decision-making.

That being said, in cases where a reversible cause of exacerbation is identified, intubation and mechanical ventilation may serve as supportive measures while targeted therapies are given time to take effect. In such instances, we suggest using low tidal volumes and low PEEP, recognizing the non-recruitable nature of ILD lungs. However, in select cases with evidence of recruitable lung units, a higher PEEP may be cautiously considered, though careful monitoring is essential to avoid complications such as barotrauma.

## 4. Other Interventions and Supportive Care

Managing ILD in the ICU requires comprehensive supportive measures tailored to their complex needs. Key interventions include antibiotic and antifibrotic therapies, fluid management, and strategies for managing pulmonary hypertension. Additionally, the role of ECMO and urgent consideration for lung transplantation are essential, highlighting the importance of a multidisciplinary approach in optimizing patient outcomes.

### 4.1. Antibiotic Therapy

Empiric antibiotics are routinely initiated in critically ill ILD patients while awaiting culture results. This is particularly important for patients who are starting high-dose corticosteroids or other immunosuppressive therapies, as these treatments increase the risk of superimposed infections. The choice of antibiotics may be informed by prior culture and sensitivity data, as well as pre-existing comorbid conditions. Careful selection of antibiotics is particularly crucial in non-IPF ILD patients who may be on chronic immunosuppressives, as they are at an increased risk of opportunistic infections [9,16]. In particular, PJP pneumonia must be considered. A review of patients with rheumatoid arthritis-associated ILD on biologic therapies revealed a high incidence of PJP, affecting 50% of the 26 patients examined [69]. Notably, PJP can present with imaging findings that mimic ILD exacerbations, making it essential to include PJP in the differential diagnosis for all ILD patients, especially those on chronic immunosuppressives. Obtaining a BAL sample, or sputum if BAL is not feasible, is critical for diagnosis. In cases where there is a delay in testing and clinical suspicion is high, empirical treatment for PJP should be initiated without waiting for confirmatory results [19,21].

Clinicians should also be aware of potential chronic or atypical infections, such as Aspergillus and non-tuberculous mycobacteria, which may require empirical treatment, especially when heavy immunosuppression is used, as these infections can lead to fulminant disease [70].

### 4.2. Antifibrotic Therapy

Antifibrotics have been shown to reduce the rate of lung function decline in both IPF and non-IPF ILD [71,72,73]. Data also demonstrate their effectiveness in reducing the rates of acute exacerbations [74]. However, these medications primarily serve a preventive function, and there are currently little to no recommendations for initiating antifibrotic therapy in patients experiencing an acute exacerbation in the ICU [75].

### 4.3. Fluid Management

The importance of maintaining a net negative or neutral fluid balance in patients with AE-ILD is being increasingly recognized [12]. Overall fluid status has also been identified as a predictor of mortality in ventilated patients with AE-ILD [6,60]. Aggressive fluid management is also essential in the management of concomitant pulmonary hypertension as right ventricular (RV) offloading may be necessary in cases of RV failure [76].

### 4.4. Management of Concomitant Pulmonary Hypertension

**Figure 5:**
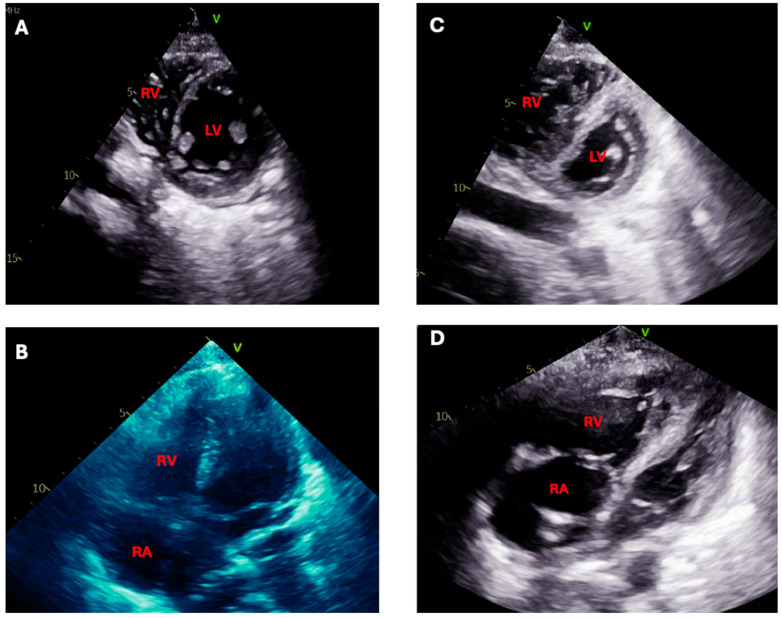
Echocardiographic images from a patient with rapidly progressive interstitial lung disease admitted to the ICU. Images (**A**,**B**), taken at initial presentation, show a normal right ventricle (RV), left ventricle (LV), and right atrium (RA). Five days later, repeat imaging reveals acute right ventricular dilation in both the parasternal short-axis (image (**C**)) and apical 4-chamber (image (**D**)) views, consistent with rapid development of right ventricular failure in the setting of ILD exacerbation.

Patients with severe ILD often have concomitant pulmonary hypertension. These patients may present with signs of RV failure as their initial presentation or develop RV failure during their ICU stay [77]. The signs of RV failure are often nonspecific, such as severe hypoxemia and hypotension, and can be confused with other potential etiologies, leading to delays in diagnosis and management. Frequent echocardiographic assessments and a high index of suspicion are required, as these patients can deteriorate rapidly [76] (Figure 5). Pulmonary hypertension is associated with a poor prognosis among patients with ILD and can be particularly detrimental when coupled with RV failure in the ICU [78,79,80,81].

Managing acute RV failure in these patients is challenging due to the pressure-sensitive nature of RV failure. Treatment may include inhaled vasodilators such as nitric oxide or epoprostenol to induce vasodilation in the ventilated units of the lungs [82,83]. Intravenous vasodilators should be used cautiously, as they can worsen ventilation/perfusion mismatch, potentially leading to worsening hypoxemia [84,85]. In terms of ventilatory strategies, high PEEP should be avoided, as it can increase intrathoracic pressure and reduce RV preload. For patients who do not respond to less invasive supportive interventions, mechanical circulatory support devices, such as veno-arterial ECMO and RV Impella, may be considered in severe cases [76].

### 4.5. Extracorporeal Membrane Oxygenation (ECMO)

In recent years, the emergence and increasing use of ECMO has revolutionized the management of respiratory failure in the ICU. In the context of ILD, ECMO offers a potential modality to support oxygenation and gas exchange while allowing for ‘lung rest’ and offering a bridge for patients awaiting lung transplantation or undergoing urgent transplant evaluation when outpatient assessment is not possible [86]. Studies have shown that ECMO is associated with high mortality in patients who are not eligible for lung transplantation compared to those eligible for transplantation. For patients on ECMO as a bridge to transplantation, survival rates are comparable to critically ill pre-transplant patients not requiring ECMO. Additionally, patients who underwent urgent transplant evaluation and listing while on ECMO had similar long-term survival and allograft function to those listed as outpatients [86,87,88]. Although ECMO has opened new avenues for managing patients with end-stage ILD, it carries serious complications; therefore, its use should be individualized and based on thorough, patient-centric multidisciplinary discussions.

### 4.6. Lung Transplantation

ILD is the leading indication for lung transplantation, which may be the only salvage option for advanced ILD patients. Typically, lung transplantation evaluation and workup are completed in the outpatient setting. However, in some cases, patients admitted to the ICU with advanced fibrosis may need urgent evaluation for transplantation. Several relative and absolute contraindications to lung transplantation are particularly relevant for ICU patients, including multiorgan failure, sepsis, and a glomerular filtration rate (GFR) <60 mL/min/1.73 m^2^ [89].

A single-center study by Dotan et al. showed that patients with *an acute exacerbation of IPF (already listed)* who underwent transplantation had worse short- and long-term survival compared to patients with stable IPF (1-year and 3-year survival rates of 94% and 90% for stable IPF vs. 71% and 60% for patients who were transplanted during AE-IPF) [90]. In contrast, a large study by Tang et al. found that patients who were *urgently listed* for lung transplantation—75% of whom had *restrictive lung disease*—had similar outcomes to those listed electively (1-year survival of 75% in the elective group vs. 78% in the urgent group) [91]. These findings suggest a hopeful outlook for patients who may otherwise have no salvageable treatment options. It also reflects improvements in our ability to provide advanced life support through mechanical ventilation, pulmonary vasodilators, and ECMO, enabling us to sustain critically ill patients longer and facilitate their eligibility for transplantation.

Despite these advancements, contraindications such as multiorgan failure, poor functional status, and the inability to participate in rehabilitation remain crucial factors when considering lung transplantation in ICU patients [92]. Aggressive efforts are being made to improve the nutrition and functional status of patients who are being considered for or are listed for lung transplantation; however, these interventions lack specific evidence supporting their effectiveness.

### 4.7. Multidisciplinary Approach

The management of patients with ILD requires the involvement of multiple specialists and services. Rheumatologists play a crucial role in diagnosing new cases of connective tissue disease-related ILD and in navigating the complex decisions regarding the administration and choice of immunosuppressive agents. Castelino et al. demonstrated that, with rheumatology input, one-third of patients in the ICU received a CTD diagnosis different from their initial referral diagnosis [93].

Similarly, palliative care is vital for patients with advanced ILD who often suffer from debilitating symptoms such as severe dyspnea, fatigue, and cough, as well as psychosocial distress due to their progressive and life-limiting condition. These patients also face high ICU-related mortality, particularly during acute exacerbations. Palliative care in ILD goes beyond end-of-life care; it involves the early management of symptoms, discussions about goals of care, and psychosocial support for both patients and their families. This is particularly important for those newly diagnosed with ILD in the ICU who may face overwhelming emotional and physical challenges. Studies have shown that early integration of palliative care into the treatment plan of patients with ILD in an outpatient setting improves symptom control, enhances quality of life, and facilitates advanced care planning, leading to better alignment between patient wishes and the care they receive [94].

Early palliative care involvement can also help in navigating challenging decisions if patients become critically ill, such as the appropriateness of invasive therapies like mechanical ventilation or ECMO in patients with advanced ILD. For example, in patients with progressive ILD, timely conversations around the goals of care can prevent unwanted interventions and focus on comfort-oriented measures if they need hospitalization or ICU stay.

## 5. Prognosis

Several studies have shown that patients with ILD requiring ICU admission face high mortality rates [2,87,95]. A systematic review by Huapaya et al. found that IPF is associated with higher mortality in the ICU setting compared to other forms of ILD [96]. Specifically, in-hospital mortality for IPF was 79% between 1993 and 2004, which decreased to 65% in 2005–2017. In contrast, non-IPF ILD patients had lower, yet still high, mortality rates of 62% in 2001–2009, which dropped to 48% in 2010–2017. This decline is attributed to the development of antifibrotic therapies, improved understanding and application of immunosuppressives, increased use of CT imaging for better diagnosis and characterization of ILD subtypes, advances in ICU management strategies, and the availability of ECMO and lung transplantation. Despite this decline in mortality, the prognosis remains poor and overall mortality remains high, along with high morbidity. Additionally, evidence suggests that patients with IPF who survive ICU admission often die within a few months after hospital discharge, indicating a high out-of-hospital mortality rate [97]. Mechanical ventilation and hypoxia during hospitalization were identified as risk factors regardless of the type of ILD [96,97].

## 6. Future Directions

A significant gap remains in our understanding of the management of ILD, particularly in the ICU setting, highlighting the need for further investigation and an individualized, evidence-based medical approach. Looking ahead, ongoing prospective randomized controlled trials, such as STRIVE-IPF [NCT03286556] and EXCHANGE_IPF (NCT03584802), are exploring the efficacy and safety of combination therapy involving plasma exchange, IVIG, and rituximab for managing AE-IPF. Another trial, EXAFIP2 (NCT05674994), is comparing the use of glucocorticoids with placebo in AE-IPF, while NCT05842681 is examining the use of azithromycin in this context. In addition to the ongoing work on management strategies, the REACT (NCT06445686) and IERATIC (NCT05313672) trials are investigating patient hemodynamics before and after an exacerbation and respiratory mechanics in patients with AE-IPF.

Furthermore, there is a growing interest in identifying biomarkers to more reliably characterize acute exacerbations across various ILDs. Biomarkers related to alveolar epithelial cells, fibroproliferation, and immune molecules are being investigated to improve our understanding of these acute events and potentially refine treatment strategies [98].

These future studies are crucial for expanding our knowledge of ILD management in the ICU, and they hold the potential to significantly improve patient outcomes.

## 7. Conclusions

Managing patients with ILD in the ICU setting requires a nuanced and multifaceted approach due to the complexity of the disease and its associated complications. Accurate diagnosis relies heavily on imaging and thorough investigations. Treatment often involves a combination of corticosteroids, cautious use of other immunosuppressive agents, and careful application of oxygenation and ventilation strategies, as well supportive ICU care. The role of multidisciplinary care, including rheumatologists and palliative care teams, is crucial in optimizing patient outcomes. For patients with advanced disease, urgent evaluation for lung transplantation, possibly with ECMO support, may be considered a lifesaving intervention. Despite advancements in life support and management strategies, the high mortality rates among patients with ILD in the ICU underscore the need for ongoing research and tailored interventions.

## Figures and Tables

**Table 1:** Common causes of acute respiratory decompensation in patients with ILD [2,12].

Causes of Acute Decompensation in Patients with ILD
1. Extra-parenchymal causes:
a. Pneumothorax
b. Pulmonary embolism
c. Pleural effusion
2. Pulmonary edema
3. Acute exacerbation of ILD (triggers: infection, occupational/environmental exposure, aspiration)
